# Shear wave elastography of transverse carpal ligament increased with simulated carpal tunnel pressure

**DOI:** 10.1186/s13018-024-04874-x

**Published:** 2024-07-02

**Authors:** Hui Zhang, John C. Elfar, C. Kent Kwoh, Zong-Ming Li

**Affiliations:** 1 Hand Research Laboratory, Department of Orthopaedic Surgery, University of Arizona College of Medicine, 1501 N Campbell Avenue, Tucson, AZ 85724 USA; 2 Arthritis Center, University of Arizona, Tucson, AZ USA; 3 Department of Biomedical Engineering, University of Arizona, Tucson, AZ USA

**Keywords:** Carpal tunnel pressure, Shear wave elastography, Transverse carpal ligament, Ultrasound imaging

## Abstract

**Background:**

Elevation of carpal tunnel pressure is known to be associated with carpal tunnel syndrome. This study aimed to correlate the shear wave elastography in the transverse carpal ligament (TCL) with carpal tunnel pressures using a cadaveric model.

**Methods:**

Eight human cadaveric hands were dissected to evacuate the tunnels. A medical balloon was inserted into each tunnel and connected to a pressure regulator to simulate tunnel pressure in the range of 0-210 mmHg with an increment of 30 mmHg. Shear wave velocity and modulus was measure in the middle of TCL.

**Results:**

SWV and SWE were significantly dependent on the pressure levels (*p* < 0.001), and positively correlated to the tunnel pressure (SWV: *R* = 0.997, *p* < 0.001; SWE: *R* = 0.996, *p* < 0.001). Regression analyses showed linear relationship SWV and pressure (SWV = 4.359 + 0.0263 * Pressure, R^2^ = 0.994) and between SWE and pressure (SWE = 48.927 + 1.248 * Pressure, R^2^ = 0.996).

**Conclusion:**

The study indicated that SWV and SWE in the TCL increased linearly as the tunnel pressure increased within the current pressure range. The findings suggested that SWV/SWE in the TCL has the potential for prediction of tunnel pressure and diagnosis of carpal tunnel syndrome.

## Introduction

Carpal tunnel syndrome is the most common peripheral neuropathy, with a prevalence rate of around 4% in the general population [1]. The syndrome is caused by compression on the median nerve, which is potentially a result of increased pressure levels inside the carpal tunnel and hardening of the transverse carpal ligament (TCL). The pressure in the tunnel of healthy subjects is less than 15 mmHg, in comparison to greater than 30 mmHg in patients with carpal tunnel syndrome [2–5]. In extreme cases, the pressure can be as high as 250 mmHg [2] and is sensitive to wrist postural changes and digit loading [5, 6]. High tunnel pressure leads to median nerve ischemia and sensory and motor changes [7, 8], causing symptoms of carpal tunnel syndrome. Investigating carpal tunnel pressure helps understand the etiology of carpal tunnel syndrome and improve its diagnosis.

Carpal tunnel pressure is measured in vivo by directly inserting a catheter sensor into the tunnel in human hands [2, 3, 5, 9]. The in-vivo use of this technique is limited because of invasiveness and the risk of injury to critical structures. Moreover, the catheter itself may interfere with the measurement, and its tip may cause damage to the median nerve and other structures [9]. No non-invasive method exists to measure tunnel pressure.

Shear wave elastography is a potential modality to non-invasively measure carpal tunnel pressure. The pressure in the carpal tunnel induces tension in the surrounding soft tissues, which might affect the shear wave propagation speed. For instance, a previous study using a cadaveric model demonstrated that shear wave speed of flexor digitorum superficialis altered with magnitude of tunnel pressure [10]. Another possible tissue to reflect tunnel pressure is the transverse carpal ligament (TCL). As the volar boundary of the tunnel, the TCL undergoes morphological changes [11–13] at different tunnel pressure levels, possibly due to the pressure caused tension stretching the TCL. Therefore, shear wave propagation in the TCL may be indicative of carpal tunnel pressure.

The purpose of this study was to investigate the changes of the shear wave velocity (SWV) and shear wave elasticity (SWE) of the TCL as carpal tunnel pressure was regulated in cadaveric hands. We hypothesized that SWV and SWE of the TCL would increase as the carpal tunnel pressure increased.

## Methods

### Specimen preparation

Eight fresh-frozen cadaveric hands (age 67.5 ± 7.0 years, height 168.6 ± 8.4 cm, weight 72.6 ± 16.2 kg, body mass index 26.1 ± 3.9 kg/m^2^) were used in the study. These specimens had no history of musculoskeletal disorders or injuries to the hand and wrist. Each specimen was thawed overnight at room temperature prior to experiment. For dissection preparation, the specimen was transected to make two 4-cm cuts: one was 1 cm proximal to the distal wrist crease, and the other 4 cm proximal to the distal wrist crease. At the two cuts, the carpal tunnel contents of the median nerve and flexor tendons were pulled out from the proximal cut to evacuate the carpal tunnel. The skin and soft tissues above the TCL remained intact.

The TCL at the middle level of the carpal tunnel was identified and marked for each specimen. Specifically, the TCL’s distal bony attachment onto the hook of hamate and ridge of trapezium was identified first, with the help of ultrasound imaging, and then an opaque skin marker (X-sports, Beekley Corp., Conn., USA) consisting of a 1.5-mm-diameter pellet was attached to the skin above each bony attachment site with the assistance of forceps. The same operations were repeated to identify and mark the TCL’s proximal bony attachment onto the pisiform and scaphoid. Based on the four skin markers, the midpoints between the markers on the hamate and pisiform, and between the markers on the trapezium and scaphoid, were found. Another two skin markers were attached to the two midpoints using Loctite Super Glue, and the previous four skin markers on the four bony attachment sites were removed thereafter. These remaining two marks on the midpoints would later assist the position the ultrasound transducer at the middle TCL and determine the radial and ulnar end points of the TCL on ultrasound image.

### Experimental apparatus

A custom pressurization system consisting of a medical balloon (Nordan Medical, Salem, NH), a pressure gauge (Dwyer Instruments, INC, Michigan City, IN, USA), and a pressure regulator (Electric Tourniquet 1000 ELC, VBM Medical Inc., Noblesville, IN, USA) was used to regulate carpal tunnel pressure with a pressure accuracy of ± 1% full-scale and regulation accuracy of ± 2–3 mmHg. The balloon was fitted on one end with the pressure regulator, and the other end sealed. An ultrasound system with a 18L6 linear array ultrasound transducer (SuperSonic MACH 30, Hologic Inc., Marlborough, MA, USA) was used to quantify the shear wave velocity. The ultrasound transducer was mounted on a positioning apparatus to fix the transducer’s position during data collection.

### Experimental procedures

**Fig. 1:**
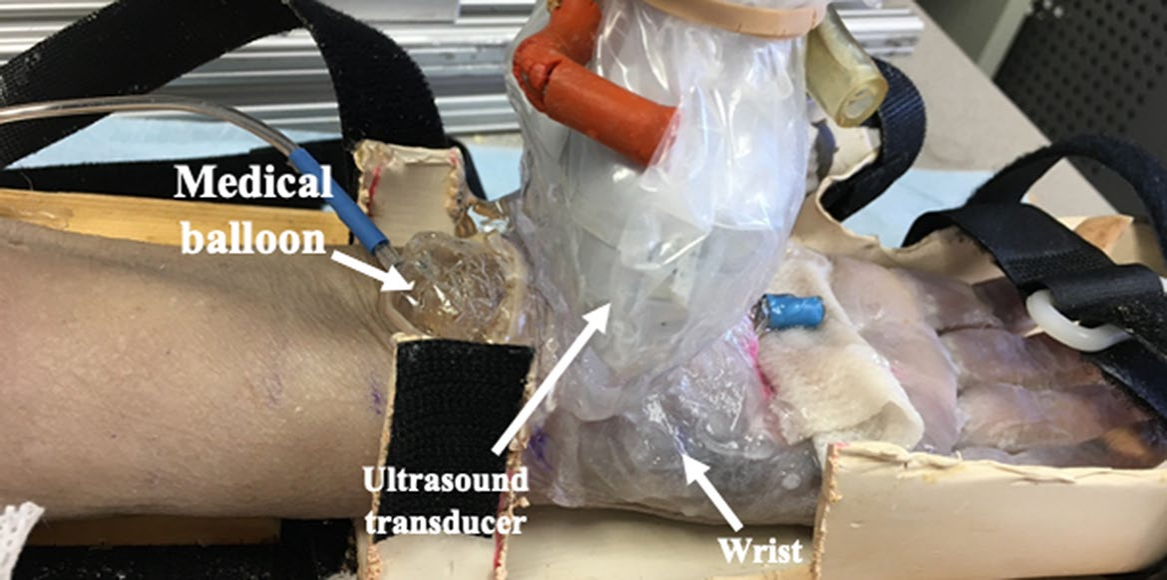
Experimental setup for carpal tunnel imaging. Note that the ultrasound transducer was encased in a specialized cover for hygiene consideration

**Fig. 2:**
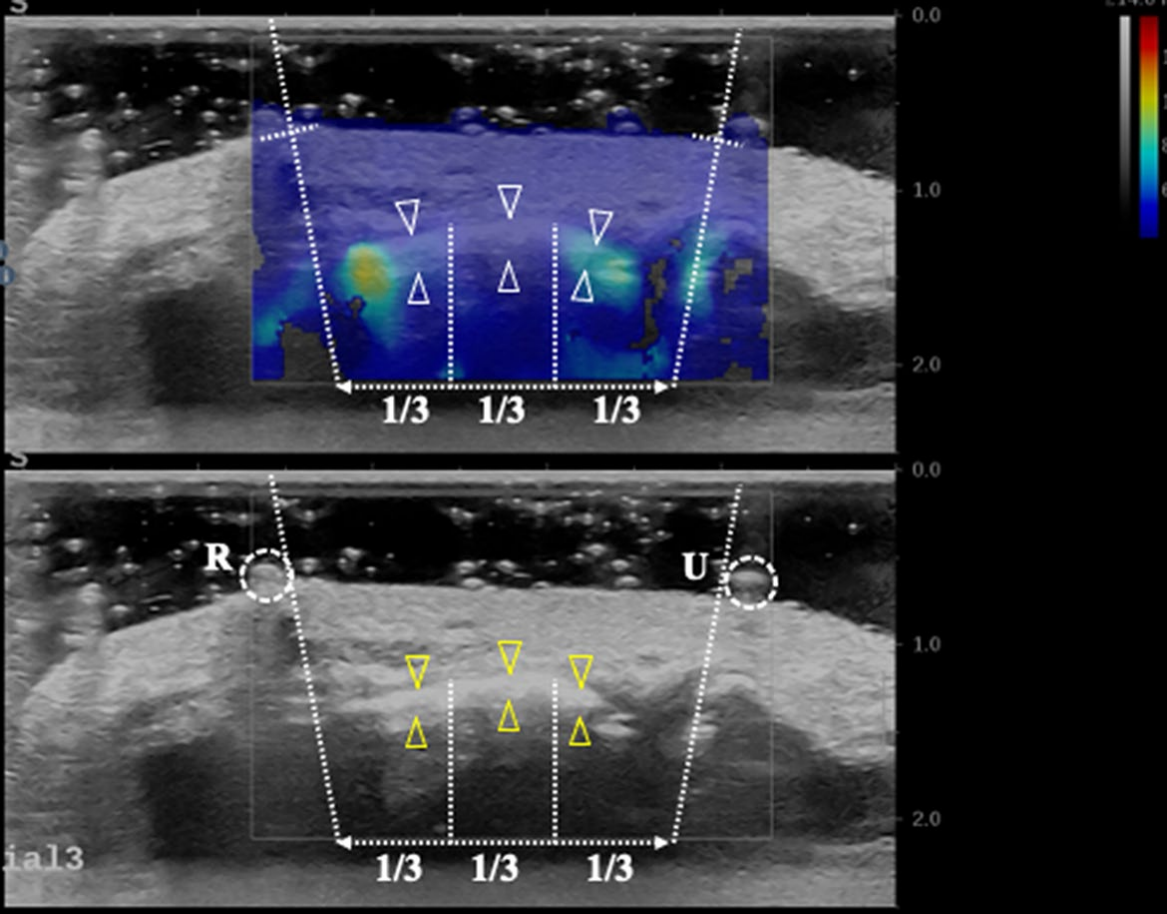
Representative ultrasound images with the outlined middle 1/3 of the transverse carpal ligament. The TCL was labeled by the triangles indicating its volar and dorsal boundaries. The circles indicated as R and U are the two opaque skin markers at radial and ulnar used to approximately defined endpoints of the TCL

The specimen was secured in a custom-made splint with the wrist in an anatomically neutral position and the thumb in radial abduction. Through the specimen’s proximal cut, the deflated balloon was inserted into the carpal tunnel and centered beneath the TCL with the help of ultrasound imaging (Fig. 1). Next, the ultrasound transducer was moved to image the TCL’s middle segment wherein both the two skin markers appeared in the ultrasound image (Fig. 2). To avoid the transducer touching the skin, a distance of 1.0 cm between the ultrasound transducer and the volar surface of the wrist was maintained, and ultrasound gel was used to fill the gap. Before data collection, the ultrasound system was set to feature “penetration” for optimization, m/s for SWV unit, and 0 ~ 14s m/s as velocity range. The imaging window’s position and size were adjusted to cover and center the TCL. For data collection, 8 different pressure levels, ranging from 0 to 210 mmHg at increments of 30 mmHg, were tested within the tunnel in a randomized order. The pressure was repeated three times, totaling 24 trials. Within each trial, the pressure regulator was used to simulate and maintain the desired pressure level in the tunnel. Then the specimen rested for one minute to minimize the viscoelastic stress relaxation before collecting shear wave elastography images. Both the B-mode and SWE images were taken. After imaging, the system pressure was released to 0 mmHg, and the specimen rested for one minute before starting a new pressure level.

### Data analyses

From the B-mode image, the TCL region, with its two ends indicated by the two skin markers, was identified and equally divided into three segments (Fig. 2). The middle segment was boxed by a rectangular with height of 1 mm, using the Q-BOX^™^ trace function. The mean SWV and SWE values were calculated by automation.

## Results

**Fig. 3:**
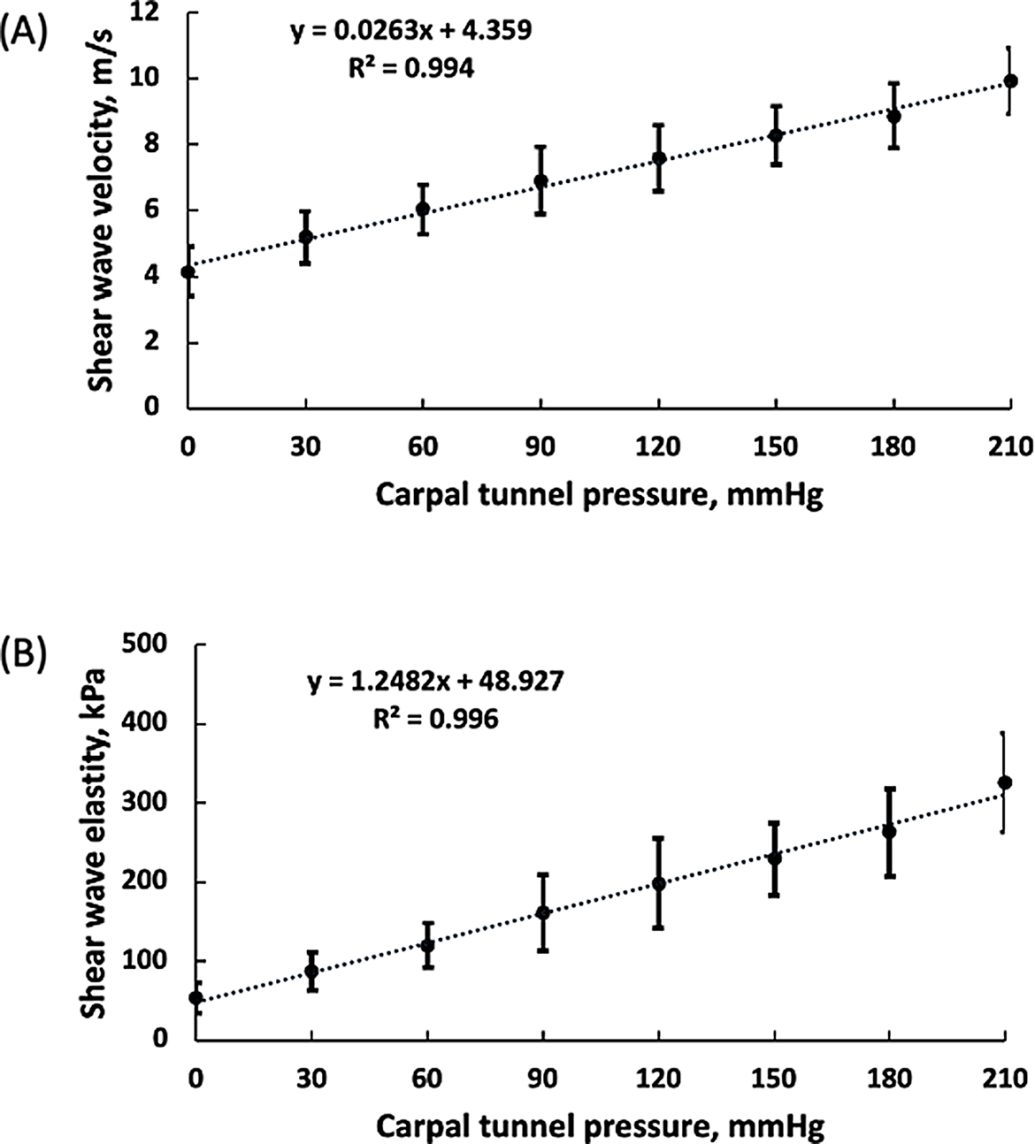
Changes of shear wave velocity (SWV) and shear wave elasticity (SWE) as carpal tunnel pressure increased (discrete data points and the regression lines). The data points are means ± standard deviations

The value of SWV at 0, 30, 60, 90, 120, 150, 180, 210 mmHg was 4.14 ± 0.75, 5.20 ± 0.78, 6.04 ± 0.75, 6.91 ± 1.02, 7.60 ± 1.01, 8.28 ± 0.89, 8.87 ± 0.97, 9.93 ± 1.01 m/s, respectively. As the tunnel pressure level increased, SWV increased (Fig. 3a). The one-way repeated measures ANOVA revealed that SWV was significantly dependent on the pressure levels (*p* < 0.001). Post hoc test for multiple comparisons found that SWV was significantly different between all the pairs (*p* < 0.05), except for 150 mmHg vs. 180 mmHg (*p* = 0.132). Pearson’s correlation coefficient to assess the linear relation between SWV and carpal tunnel pressure showed that there was a positive correlation between the two variables (*R* = 0.997, *p* < 0.001). Linear regression showed that the fitted regression model was SWV = 4.359 + 0.0263 * Pressure (R^2^ = 0.994).

The value of SWE at 0, 30, 60, 90, 120, 150, 180, 210 mmHg was 54.67 ± 19.31, 87.03 ± 23.88, 120.09 ± 27.64, 161.37 ± 47.90, 198.29 ± 56.58, 229.17 ± 46.33, 262.86 ± 55.84, 326.39 ± 62.67 kPa, respectively. Similar to SWV results, as the tunnel pressure level increased, SWE increased (Fig. 3b). The one-way repeated measures ANOVA revealed that SWE was significantly dependent on the pressure levels (*p* < 0.001). Post hoc test for multiple comparisons found that the mean value of SWV was significantly different between all the pairs (*p* < 0.05), except for the pairs 120 mmHg vs. 150 mmHg (*p* = 0.161) and 150 mmHg vs. 180 mmHg (*p* = 0.095). Pearson’s correlation coefficient to assess the linear relation between SWE and carpal tunnel pressure showed that there was a positive correlation between the two variables (*r* = 0.996, *p* < 0.001). Linear regression showed that the fitted regression model was: SWE = 48.927 + 1.248 * Pressure (R^2^ = 0.996).

## Discussion

Shear wave elastography has been widely used to investigate diverse human organs and tissues, including liver [14], breast [15], kidney [16], spleen [17], prostate [18], bladder [19], pancreas [20], testes [21], thyroid [22], muscle, and tendon [23–27]. Recently, SWE has been used to examine tissues related to the carpal tunnel. For example, it was reported that elastography of the median nerve itself can be used to differentiate neuropathy in individuals with carpal tunnel syndrome [28–31]. However, the median nerve is not released to treat carpal tunnel syndrome, and this work has not previously focused on the target of surgical treatment. Other studies showed that SWE of flexor tendons inside the carpal tunnels of turkeys [32] and humans [10], are correlated with tunnel pressure. Here again, the most superficial and accessible part of the carpal tunnel, and indeed the part that is released during surgery was not the focus of this work.

We demonstrated that shear wave elastography of TCL could be potentially predict pressure in the carpal tunnel by measuring a pathological finding in the actual ligament that is released at surgery. In this study, we used a cadaveric model to evaluate the relation between SWV/SWE in the TCL and precise carpal tunnel pressure. Different carpal tunnel pressure levels were simulated by regulating the air inflation of the medical balloon that was inserted into the carpal tunnel of cadaver hands. The corresponding SWV/SWE in the middle portion of the TCL was measured by shear wave elastography. We found SWV/SWE in the TCL was closely associated with carpal tunnel pressure. Within the current range of tunnel pressure, SWV/SWE increased linearly with the carpal tunnel pressure level. The observation suggested that SWV/SWE in the TCL might be useful to measure the pressure level in the carpal tunnel non-invasively.

In this study, the increase of SWV/SWE could result from increased tension in the TCL due to pressure in the carpal tunnel. According to Laplace law, pressure inside a cylindrical membrane generates surface tension – the greater the pressure, the greater the tension. In the cylinder-like carpal tunnel, pressure would cause tensile stress and strain of the volarly situated TCL. Literature has shown shear wave elastography can be used as a non-invasive method to correlate tendon and muscle forces [23, 24]. For example, a previous study reported tendon forces can be measured as a linear function of SWV in a cadaveric patellar tendon model. Bouillard et al. (2012) showed that SWE of first dorsal interosseous and abductor digiti minimi was linearly related to the torque generation by the muscles.

The changes to the shear wave propagation in the TCL could also be attributed to tension-induced fiber realignment in the TCL when it is under tension. Previous studies found that the TCL is not a uniform structure, but rather has collagen fiber bundles oriented in different directions [33, 34]. Specifically, the dominant fiber direction is transverse but these fibers are intertwined with others in the oblique direction towards the pisiform-trapezium and others in the scaphoid-hamate direction [33, 34]. When the ligament or tendon is under tension, the orientation angle of some fibers in the oblique directions may be offset, or inclined toward the loading direction [35–37]. Fiber alignment in the direction of the shear wave propagation could facilitate wave propagation resulting in higher SWV [38]. In our study, it is likely that as the carpal tunnel pressure is elevated, more and more collagen fibers in the TCL realigned from oblique direction to the transverse direction.

The findings of the linear relationship between SWV/SWE and carpal tunnel pressure should be interpreted within specific regions of the TCL. A previous study demonstrated that the thickness and mechanical modulus of the TCL are region-dependent, varying from radial to ulnar and from proximal to distal [39]. Moreover, the unevenness becomes further complicated due to thenar muscle attachments, where the TCL becomes thicker and stiffer. Shen et al. used acoustic radiation force impulse imaging to reveal greater SWV values within the muscle-attached region than those outside of the muscle-attached region [40]. To avoid the variability caused by the boundary condition, we restricted the region of interest in the middle part of the middle cross section of the TCL to obtain the robust, and clinically reproducible measurement when applied to future clinical studies.

Sex is expected to play to role in the relation between SWV/SWE and carpal tunnel pressure, but our study used only female cadaver hands. It is well known that females have a higher prevalence of CTS. Sex-related differences of tissue mechanics have been reported, including mechanical properties [41–43] and carpal arch morphology [44, 45] of the TCL. Previous studies reported that female TCLs are less elastic [41, 46] and less compliant [42] and a trend of being stiffer [43] than males. If this difference can explain the difference in prevalence between sexes, we may be able to address that in clinical translation with future studies directed at sex differences of the SWV-pressure relationship.

In conclusion, the ligament most commonly released to treat CTR shows changes documentable using a relatively non-invasive technique when tested using cadaveric specimens to evaluate the relation between SWV and SWV and carpal tunnel pressure. TCL SWV and SWE was linearly increased as the tunnel pressure increased in the physiological plausible range from 0 to 210 mmHg – findings that underline a potential non-invasive diagnostic for the key pathological entity in CTS at the site of surgical release.

## Data Availability

No datasets were generated or analysed during the current study.

## References

[1] Atroshi I, Gummesson C, Johnsson R, Ornstein E, Ranstam J, Rosen I (1999). Prevalence of carpal tunnel syndrome in a general population. JAMA.

[2] Gelberman RH, Hergenroeder PT, Hargens AR, Lundborg GN, Akeson WH (1981). The carpal tunnel syndrome. A study of carpal canal pressures. J Bone Joint Surg Am Vol.

[3] Okutsu I, Ninomiya S, Hamanaka I, Kuroshima N, Inanami H (1989). Measurement of pressure in the carpal canal before and after endoscopic management of carpal tunnel syndrome. J Bone Joint Surg Am Vol.

[4] Rojviroj S, Sirichativapee W, Kowsuwon W, Wongwiwattananon J, Tamnanthong N, Jeeravipoolvarn P (1990). Pressures in the carpal tunnel. A comparison between patients with carpal tunnel syndrome and normal subjects. J Bone Joint Surg Br Vol.

[5] Weiss ND, Gordon L, Bloom T, So Y, Rempel DM (1995). Position of the wrist associated with the lowest carpal-tunnel pressure: implications for splint design. J Bone Joint Surg Am.

[6] Rempel D, Keir PJ, Smutz WP, Hargens A (1997). Effects of static fingertip loading on carpal tunnel pressure. J Orthop Res.

[7] Diao E, Shao F, Liebenberg E, Rempel D, Lotz JC (2005). Carpal tunnel pressure alters median nerve function in a dose-dependent manner: a rabbit model for carpal tunnel syndrome. J Orthop Res.

[8] Lundborg G, Gelberman RH, Minteer-Convery M, Lee YF, Hargens AR (1982). Median nerve compression in the carpal tunnel—functional response to experimentally induced controlled pressure. J Hand Surg.

[9] Luchetti R, Schoenhuber R, De Cicco G, Alfarano M, Deluca S, Landi A (1989). Carpal-tunnel pressure. Acta Orthop Scand.

[10] Kubo K, Zhou B, Cheng YS (2018). Ultrasound elastography for carpal tunnel pressure measurement: a cadaveric validation study. J Orthop Res.

[11] Mesgarzadeh M, Schneck CD, Bonakdarpour A, Mitra A, Conaway D (1989). Carpal tunnel: MR imaging. Part II. Carpal tunnel syndrome. Radiology.

[12] Monagle K, Dai G, Chu A, Burnham R, Snyder R (1999). Quantitative MR imaging of carpal tunnel syndrome. AJR Am J Roentgenol.

[13] Tsujii M, Hirata H, Morita A, Uchida A (2009). Palmar bowing of the flexor retinaculum on wrist MRI correlates with subjective reports of pain in carpal tunnel syndrome. J Magn Reson Imaging.

[14] Palmeri ML, Wang MH, Dahl JJ, Frinkley KD, Nightingale KR (2008). Quantifying hepatic shear modulus in vivo using acoustic radiation force. Ultrasound Med Biol.

[15] Meng W, Zhang G, Wu C, Wu G, Song Y, Lu Z (2011). Preliminary results of acoustic radiation force impulse (ARFI) ultrasound imaging of breast lesions. Ultrasound Med Biol.

[16] Stock KF, Klein BS, Vo Cong MT (2010). ARFI-based tissue elasticity quantification in comparison to histology for the diagnosis of renal transplant fibrosis. Clin Hemorheol Microcirc.

[17] Bota S, Sporea I, Sirli R, Popescu A, Danila M, Sendroiu M, Focsa M (2010). Spleen assessment by Acoustic Radiation Force Impulse Elastography (ARFI) for prediction of liver cirrhosis and portal hypertension. Med Ultrason.

[18] Zhai L, Polascik TJ, Foo WC, Rosenzweig S, Palmeri ML, Madden J, Nightingale KR (2012). Acoustic radiation force impulse imaging of human prostates: initial in vivo demonstration. Ultrasound Med Biol.

[19] Sturm RM, Yerkes EB, Nicholas JL (2017). Ultrasound Shear Wave Elastography: a Novel Method to evaluate bladder pressure. J Urol.

[20] D’Onofrio M, Gallotti A, Martone E, Pozzi Mucelli R (2009). Solid appearance of pancreatic serous cystadenoma diagnosed as cystic at ultrasound acoustic radiation force impulse imaging. JOP.

[21] D’Anastasi M, Schneevoigt BS, Trottmann M, Crispin A, Stief C, Reiser MF, Clevert DA (2011). Acoustic radiation force impulse imaging of the testes: a preliminary experience. Clin Hemorheol Microcirc.

[22] Friedrich-Rust M, Romenski O, Meyer G (2012). Acoustic Radiation Force impulse-imaging for the evaluation of the thyroid gland: a limited patient feasibility study. Ultrasonics.

[23] Ahmadzadeh SMH, Chen X, Hagemann H, Tang MX, Bull AMJ (2019). Developing and using fast shear wave elastography to quantify physiologically-relevant tendon forces. Med Eng Phys.

[24] Bouillard K, Nordez A, Hug F (2011). Estimation of individual muscle force using elastography. PLoS ONE.

[25] Corrigan P, Zellers JA, Balascio P, Silbernagel KG, Cortes DH (2019). Quantification of Mechanical properties in Healthy Achilles Tendon using continuous Shear Wave Elastography: a reliability and validation study. Ultrasound Med Biol.

[26] Kot BC, Zhang ZJ, Lee AW, Leung VY, Fu SN (2012). Elastic modulus of muscle and tendon with shear wave ultrasound elastography: variations with different technical settings. PLoS ONE.

[27] Payne C, Watt P, Cercignani M, Webborn N (2018). Reproducibility of shear wave elastography measuresof the Achilles tendon. Skeletal Radiol.

[28] Cingoz M, Kandemirli SG, Alis DC, Samanci C, Kandemirli GC, Adatepe NU (2018). Evaluation of median nerve by shear wave elastography and diffusion tensor imaging in carpal tunnel syndrome. Eur J Radiol.

[29] Ibrahim HR. Diagnostic value of median nerve shear wave ultrasound elastography in diagnosis and differentiation of carpal tunnel syndrome severity. Egypt J Radiol Nuclear Med. 2021;52. 10.1186/s43055-021-00573-3.

[30] Kantarci F, Ustabasioglu FE, Delil S (2014). Median nerve stiffness measurement by shear wave elastography: a potential sonographic method in the diagnosis of carpal tunnel syndrome. Eur Radiol.

[31] Sung JH, Kwon YJ, Baek SH, Son MH, Lee JH, Kim BJ (2022). Utility of shear wave elastography and high-definition color for diagnosing carpal tunnel syndrome. Clin Neurophysiol.

[32] Toyoshima Y, Webb J, Gregory A, Fatemi M, Alizad A, Zhao C (2020). Ultrasound shear wave elastography for measuring intracompartmental pressure of compartment syndrome using a Turkey Hind limb model. J Biomech.

[33] Isogai S, Murakami G, Wada T, Akita K, Yamashita T, Ishii S (2002). Laminar configuration of the transverse carpal ligament. J Orthop Sci.

[34] Prantil RK, Xiu K, Kim KE, Gaitan DM, Sacks MS, Woo SL, Li ZM (2012). Fiber orientation of the transverse carpal ligament. Clin Anat.

[35] Hansen KA, Weiss JA, Barton JK (2002). Recruitment of tendon crimp with applied tensile strain. J Biomech Eng.

[36] Lake SP, Cortes DH, Kadlowec JA, Soslowsky LJ, Elliott DM (2012). Evaluation of affine fiber kinematics in human supraspinatus tendon using quantitative projection plot analysis. Biomech Model Mechanobiol.

[37] Lake SP, Miller KS, Elliott DM, Soslowsky LJ (2009). Effect of fiber distribution and realignment on the nonlinear and inhomogeneous mechanical properties of human supraspinatus tendon under longitudinal tensile loading. J Orthop Res.

[38] Blank JL, Thelen DG, Allen MS, Roth JD (2022). Sensitivity of the shear wave speed-stress relationship to soft tissue material properties and fiber alignment. J Mech Behav Biomed Mater.

[39] Holmes MW, Howarth SJ, Callaghan JP, Keir PJ (2012). Biomechanical properties of the transverse carpal ligament under biaxial strain. J Orthop Res.

[40] Shen ZL, Vince DG, Li ZM (2013). In vivo study of transverse carpal ligament stiffness using acoustic radiation force impulse (ARFI) imaging. PLoS ONE.

[41] Brett AW, Oliver ML, Agur AM, Edwards AM, Gordon KD (2014). Quantification of the transverse carpal ligament elastic properties by sex and region. Clin Biomech (Bristol Avon).

[42] Li ZM (2005). Gender difference in carpal tunnel compliance. J Musculoskelet Res.

[43] Mathers B, Agur A, Oliver M, Gordon K (2016). Biaxial quantification of deep layer transverse carpal ligament elastic properties by sex and region. Clin Biomech (Bristol Avon).

[44] Lakshminarayanan K, Shah R, Li ZM (2019). Sex-related differences in carpal arch morphology. PLoS ONE.

[45] Sassi SA, Giddins G (2016). Gender differences in carpal tunnel relative cross-sectional area: a possible causative factor in idiopathic carpal tunnel syndrome. J Hand Surg Eur Vol.

[46] Lin R, Lin E, Engel J, Bubis J (1983). Histo-mechanical aspects of carpal tunnel syndrome. Hand.

